# 71. Diagnostic Stewardship of *Clostridioides difficile* Testing

**DOI:** 10.1093/ofid/ofab466.273

**Published:** 2021-12-04

**Authors:** Zahra Qamar, Lisa A Spacek, Dagan Coppock, Kaya Patel, Nathan L’Etoile, Jingwen Huang, Akanksha Arya, Phyllis Flomenberg

**Affiliations:** 1 Thomas Jefferson University Hospital, Jenkintown, Pennsylvania; 2 Thomas Jefferson University Hospital/Sidney Kimmel Medical College of Thomas Jefferson University, Philadelphia, Pennsylvania; 3 Thomas Jeffeson University Hospital, Atlanta, Georgia

## Abstract

**Background:**

*C. difficile* (CD) testing is frequently ordered inappropriately. Highly sensitive polymerase chain reaction (PCR) tests can detect CD colonization leading to misdiagnosis. Providers often overlook other causes of diarrhea, notably laxatives. To improve diagnostic stewardship, our hospital introduced an electronic medical record (EMR)-based order set (OS).

**Methods:**

In a 926-bed, teaching hospital, we conducted a 3-step intervention to improve CD diagnostic stewardship. (1) A retrospective analysis of CD orders before and after OS implementation was done to assess its impact on inappropriate orders. The OS included two questions: (a) Did patient have ≥ 3 loose bowel movements in past 24 hours? and (b) No laxatives in past 24 hours? An appropriate order was defined if “yes” to both questions. It was still appropriate if “no” to either question but ≥ 2 unexplained following features: fever > 100.4 F, abdominal pain, megacolon, ileus or leukocytosis > 11,000 cells/mm^3^ in prior day. (2) After implementation of OS, house staff compliance with OS was surveyed via email. (3) Rationale for inappropriate orders was discussed with providers.

**Results:**

Of 238 patients in retrospective analysis, 44% were ≥ 65 years and 37% had other potential causes of diarrhea. Common clinical features were leukocytosis (40%) and fever (31%). There was no significant difference in inappropriate testing: pre-OS 27/99 (27%) vs post-OS 44/139 (32%) (p=0.47). Of 43 house officers who participated in the survey, 75% indicated they over rode the OS. When asked to provide rationale of inappropriate CD testing, providers acknowledged inappropriate ordering but did not want to miss a CD diagnosis and frequently overlooked other causes of diarrhea.

**Conclusion:**

Appropriate CD testing relies on providers’ appreciation of a clinical picture consistent with CD infection, confirmation of clinically significant diarrhea, and consideration of other causes of diarrhea. Providers order inappropriate tests, not due to lack of knowledge, but likely fear of missing diagnosis and overlooking other causes of diarrhea.

**Disclosures:**

**All Authors**: No reported disclosures

